# Factors Affecting the Quality of Person-Generated Wearable Device Data and Associated Challenges: Rapid Systematic Review

**DOI:** 10.2196/20738

**Published:** 2021-03-19

**Authors:** Sylvia Cho, Ipek Ensari, Chunhua Weng, Michael G Kahn, Karthik Natarajan

**Affiliations:** 1 Department of Biomedical informatics Columbia University New York, NY United States; 2 Data Science Institute Columbia University New York, NY United States; 3 Department of Pediatrics University of Colorado Anschutz Medical Campus Denver, CO United States

**Keywords:** patient generated health data, data accuracy, data quality, wearable device, fitness trackers, mobile phone

## Abstract

**Background:**

There is increasing interest in reusing person-generated wearable device data for research purposes, which raises concerns about data quality. However, the amount of literature on data quality challenges, specifically those for person-generated wearable device data, is sparse.

**Objective:**

This study aims to systematically review the literature on factors affecting the quality of person-generated wearable device data and their associated intrinsic data quality challenges for research.

**Methods:**

The literature was searched in the PubMed, Association for Computing Machinery, Institute of Electrical and Electronics Engineers, and Google Scholar databases by using search terms related to wearable devices and data quality. By using PRISMA (Preferred Reporting Items for Systematic Reviews and Meta-Analyses) guidelines, studies were reviewed to identify factors affecting the quality of wearable device data. Studies were eligible if they included content on the data quality of wearable devices, such as fitness trackers and sleep monitors. Both research-grade and consumer-grade wearable devices were included in the review. Relevant content was annotated and iteratively categorized into semantically similar factors until a consensus was reached. If any data quality challenges were mentioned in the study, those contents were extracted and categorized as well.

**Results:**

A total of 19 papers were included in this review. We identified three high-level factors that affect data quality—device- and technical-related factors, user-related factors, and data governance-related factors. Device- and technical-related factors include problems with hardware, software, and the connectivity of the device; user-related factors include device nonwear and user error; and data governance-related factors include a lack of standardization. The identified factors can potentially lead to intrinsic data quality challenges, such as incomplete, incorrect, and heterogeneous data. Although missing and incorrect data are widely known data quality challenges for wearable devices, the heterogeneity of data is another aspect of data quality that should be considered for wearable devices. Heterogeneity in wearable device data exists at three levels: heterogeneity in data generated by a single person using a single device (within-person heterogeneity); heterogeneity in data generated by multiple people who use the same brand, model, and version of a device (between-person heterogeneity); and heterogeneity in data generated from multiple people using different devices (between-person heterogeneity), which would apply especially to data collected under a bring-your-own-device policy.

**Conclusions:**

Our study identifies potential intrinsic data quality challenges that could occur when analyzing wearable device data for research and three major contributing factors for these challenges. As poor data quality can compromise the reliability and accuracy of research results, further investigation is needed on how to address the data quality challenges of wearable devices.

## Introduction

### Emerging Biomedical Data—Person-Generated Wearable Device Data

With the recent movement toward people (patient)-centered care and the widespread routine use of devices/technologies, person-generated health data (PGHD) have emerged as a promising data source for biomedical research [1]. A survey conducted in 2019 reported that 38% of Americans currently use technologies such as mobile apps or wearables to track their health data, and 28% have used them in the past [2]. Examples of PGHD include data collected passively through sensors, such as step count, heart rate, and sleep quality; data entered directly by people, such as diet, stress levels, and quality of life; and social or financial information that is not specifically health related but could potentially provide health-related insights [3]. Among the different PGHD, data generated through wearable devices are unique in that they are passively, continuously, and objectively collected in free-living conditions; such data are different from those generated through other technologies that require the manual input of data (eg, dietary tracking mobile apps) [4-7]. Therefore, person-generated wearable device data are becoming a valuable resource for biomedical researchers to provide a more comprehensive picture of the health of individuals and populations.

### Use of Person-Generated Wearable Device Data for Research Purposes

There are two ways to use wearable device data for research purposes. Typically, researchers collect wearable device data for a specific research by recruiting eligible participants and asking them to use the device for a certain period. For example, Lim et al [8] issued Fitbit devices to 233 participants and asked them to use the device for 5 days. Collecting data with this traditional method can be beneficial in that people can collect data that fits their needs, but it can be costly to recruit and follow a large number of participants for an extended period.

Researchers can also reuse existing data, which is a timely and cost-effective way to conduct research. Previous studies have used existing wearable device data collected for other research studies for their own research [8,9]. For example, McDonald et al [9] used a data set collected as part of the SingHEART/Biobank study to investigate the association between sleep and body mass index. In addition, Cheung et al [10] used data collected from a study by Burg et al [11] to develop a novel methodology to reduce the dimension of data while maintaining core information.

More recently, real-world wearable device data collected through routine use of devices have been reused for research purposes [7,12,13]. For example, the All of Us research program, which is the precision medicine initiative launched by the National Institutes of Health (NIH), initiated a Fitbit Bring-Your-Own-Device project, which allows participants to connect their Fitbit account to share data, such as physical activity, sleep, and heart rate [14]. In addition, multiple studies have shown the potential of routinely collected wearable device data for use in large-scale longitudinal multinational studies. Menai et al [15] used Withings Pulse activity tracker data of 9238 adults from 37 countries collected from 2009 to 2013 to examine the association between step counts and blood pressure. Kim et al [16] used data of more than 50,000 individuals from 185 countries collected over a month, with nearly 17 million measurements generated by Nokia Health Wireless blood pressure monitors to characterize blood pressure variability. These studies underscore the potential secondary uses of person-generated wearable device data for generating health insights from large real-world population that might not have been possible using traditional methods of data collection. Furthermore, the studies demonstrate how wearable device data add value by expanding the scope of biomedical research that can be conducted, which would not have been feasible if relying on electronic health record (EHR) data alone.

### Data Quality Challenges in the Use of Person-Generated Wearable Device Data

Data used in research studies, even data originally collected to support research, may not meet the ideal level of quality [13,17,18]. For instance, data collected daily through consumer wearables are meant to be used for routine use of devices rather than for research. Therefore, although the quality of collected data may be sufficient for an individual’s health management, it may be insufficient for research purposes. Hicks et al [19] presented the best practices for reusing large-scale consumer wearable device data that were collected through routine use. The study describes challenges with data quality, such as missing data or inaccuracy of sensor data, as these data are collected from individuals through their daily use of wearables (not through a research study). Thus, as recommended for the use of any data set, the study recommends assessing the quality of wearable device data set before conducting research. Once the research question and data set to be analyzed are identified, it is important to assess its fitness-for-use to ensure that it would produce valid analytical results that answer the research question [19].

There have been previous efforts to understand the data quality challenges for wearable device data. For example, Codella et al [7] identified the data quality dimensions that influence the analysis of PGHD. The concerns and expectations of PGHD stakeholders were identified through a literature review and mapped to the relevant data quality dimensions of an established framework [7]. However, the review does not systematically provide the steps of how they screened and selected the literature and what information they extracted within the studies. Another systematic review by Abdolkhani et al [20] identified factors influencing the quality of medical wearable device data and their corresponding dimensions from the literature. However, this review did not include literature on data from nonmedically approved wearables (eg, consumer wearable devices). As such, there is a research gap in understanding data quality challenges that arise from consumer wearables, specifically those from passively collected data, as there might be unique quality challenges associated with these types of data.

### Objectives

While assessing data quality, having a full understanding of the types of data quality challenges and the factors associated with them can be useful in implementing additional analytic procedures to ameliorate potential negative impacts or false conclusions. However, one of the barriers is that there is a lack of studies investigating the data quality challenges of wearable device data specifically for research purposes. Therefore, this study aims to (1) identify factors influencing the quality of person-generated wearable device data and potential intrinsic data quality challenges (data quality in its own right or, in other words, data quality challenges inherent to the data itself) for research, and (2) discuss implications for the appropriate use of person-generated wearable device data for research purposes based on the findings [21].

## Methods

### Data Sources and Search Strategy

We performed a rapid review following the PRISMA (Preferred Reporting Items for Systematic Reviews and Meta-analyses) guidelines. The literature search was conducted in four scholarly databases (PubMed, Association for Computing Machinery [ACM] Digital Library, Institute of Electrical and Electronics Engineers [IEEE], and Google Scholar) in June 2019. In PubMed, we used a combination of MeSH terms and keywords related to wearable devices and data quality. Terms related to mobile health were not searched because they include mobile apps or telemedicine, although the scope of this review focused specifically on passively collected data through wearable devices. The search results were limited to studies published within the past 5 years, studies conducted with human species and studies written in English language. The search was limited to 2014 onward because the characteristics of devices may change with advances in technologies, and this may result in changes in data quality challenges. Thus, the search was focused on recent publications using the year with the largest increase in the emergence of new consumer fitness trackers as a heuristic cutoff for determining recent studies [12]. The publications were sorted by best match, which is appropriate for searching studies that meet the informational needs on a topic [22].

In the ACM Digital Library and IEEE Xplore Digital Library, we used a query that combined search terms related to data quality and wearable devices. The search results were limited to studies published since 2014. To complement the search results from the 3 scholarly databases, we performed an additional literature search on Google Scholar. In total, 4 searches were conducted using different queries. The search excluded patents and citations, examined studies published since 2014, and sorted the results by relevance. Although all of the search results were reviewed for other scholarly databases, only the first 100 results for each of the 4 queries in Google Scholar were reviewed. To prevent the filter bubble effect, which customizes search results based on the search history of users, Google accounts were logged out when conducting the literature search [23]. The full query used in each database can be found in Table S1 in Multimedia Appendix 1.

### Literature Selection

Inclusion criteria were as follows: (1) papers that contained content on the data quality of wearable devices or sensor data; (2) papers that demonstrated the scope of wearable devices, including devices such as fitness trackers, sleep monitors, continuous glucose monitors, and remote blood pressure trackers; (3) papers on research-grade and consumer-grade devices; and (4) not only peer-reviewed studies, but also conference proceedings and book chapters to expand the search space.

Although smartphones can passively collect health data, studies that exclusively focused on smartphones were excluded, as they are not worn on the body. In addition, as we were interested in passively collected person-generated wearable device data being used for research, studies were excluded if (1) the study was on wearable device data that were generated by providers in a clinical setting (eg, device being used for clinician or surgical training), (2) the study was on wearable device data being used for clinical care of patients, and (3) the study was on data that were manually recorded (eg, food logging by user). Device validation studies such as testing the accuracy, reliability, or validity of the device were also excluded, as those studies were about testing the accuracy of the device rather than conducting analyses on data.

One reviewer (SC) screened the retrieved literature based on the title and abstract. After filtering based on titles and abstracts, the full text of the remaining studies was reviewed based on the same selection criteria by two reviewers (SC and KN). The reviewers discussed any discrepancies to reach a consensus on the final set of studies. The literature selection process was conducted using Covidence (Veritas Health Innovation), which is a web-based systematic review production tool.

### Data Extraction and Categorization

Overall, two reviewers (SC and KN) examined the papers to extract sentences about the factors affecting data quality. Although our focus was on wearable device data, sentences that apply to both mobile app and wearable device data were extracted as long as the content did not exclusively apply to mobile app data. The reviewers extracted the sentences and annotated the relevant factors. In addition, intrinsic data quality challenges associated with those factors were extracted if any were mentioned. Microsoft Excel was used to manage qualitative data. Codes were assigned to phrases that indicated factors influencing data quality by 1 reviewer (SC). Coded concepts were reviewed, and semantically similar concepts were consolidated into the same category. The categories were iteratively refined to derive core categories. The categories were then iteratively reviewed by domain experts (one data quality expert [KN] and one wearable device expert [IE]) to refine and validate the results. Domain experts commented on whether they agreed with the categorization and names used for each category. The discussion continued until a consensus among the reviewers and domain experts was reached.

## Results

### Literature Search and Selection Results

**Figure 1:**
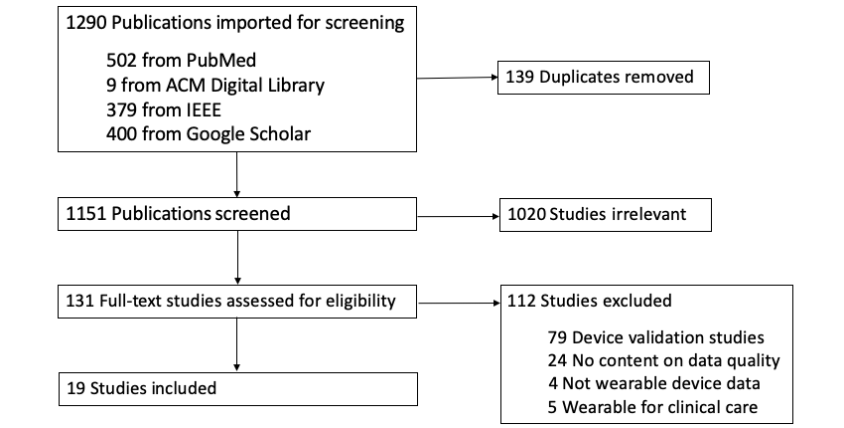
Flow diagram of the literature selection process. ACM: Association for Computing Machinery; IEEE: Institute of Electrical and Electronics Engineers.

A total of 1290 publications were retrieved for screening. Among the retrieved publications, 139 duplicates were removed, leaving 1151 unique publications to be screened by title and abstract. The screening of titles and abstracts resulted in 131 studies after removing 1020 publications that did not meet the eligibility criteria. The full texts of the remaining 131 publications were reviewed. After removing 112 irrelevant publications, 19 studies remained. The literature selection process is depicted in Figure 1, and a summary of the included studies can be found in Table S2 in Multimedia Appendix 1.

### Data Extraction and Categorization Results

Some extracted sentences were specifically related to wearable device data. For instance, sentences within a study by Wright et al [24] describe the challenges associated with using consumer fitness trackers in biomedical research:

The algorithms used in consumer physical activity monitors to determine steps taken, distance traveled, and energy expenditure are typically not shared with researchers due to proprietary concerns.

On the other hand, there were sentences that could apply to both wearable devices and mobile apps. For example, Bietz et al [25] examined data quality challenges of routine use of devices data and explicitly stated the challenges that researchers face:

Researchers also reported being concerned with the kinds of data they may get from companies, including the lack of standardization, potential problems with proprietary algorithms, and that most of the consumer-level health devices have not gone through a validation process.

Not all concerns regarding wearable device data were extracted from these studies. For example, Bietz et al [25] mentioned selection bias, which was not extracted, as we believe that bias is not an intrinsic data quality challenge but is a byproduct of data quality and a universal challenge to research design:

A related concern is the potential bias in PGHD that derives from who uses personal health devices and who does not.

After 5 iterations of categorizing the factors influencing data quality with domain experts, 3 broad categories emerged, which are summarized in Textbox 1. The mappings between the factors and the intrinsic data quality challenges are presented in Table 1.

**Factors influencing data quality and the themes identified in selected literature.**

**Device- and technical-related factors**

- Hardware issues [26-28]
  Malfunction [26,29-32]
  Quality of sensor [3,7,24,32-34]
  Sensor degradation over time [27]
  Device update makes older models outdated [24]
  Limited storage space [32]
- Software issues [24,25,27,29,34,35]
  Quality (accuracy) of algorithm [7,31,33]
  Proprietary algorithm or system [25,27,29,35]
  Wearable device companies change and update their algorithms [24]
  Software updates may change settings to default setting or affect data [34]
- Network and Bluetooth issues [29-31,34,36]
  Lost satellite connection [29,30,32,34,36]
  Delay and error in synchronization and data upload [29,30,34,36]

**User-related factors**

- User nonwear [7,24,26,30,33,34,36]
  Forget to wear [26,33]
  Nonwear during battery charging [7,24,30,34,36]
  User’s health condition prevents device use [30]
  Discomfort of wearing the device [7,24]
  Unsatisfied with the appearance of device [30]
  User’s lifestyle or not wearing for certain everyday activities [30]
  Concerns over privacy and security of data [30]
  Poor usability experience [30]
- User error [27,29-31,33,34,37]
  Device not synced by users [29]
  Poor calibration of the device [37]
  Quality of skin contact [34]
  Misplacement of device on the body [24,27,34]

**Data governance-related factors**

- Lack of standardization [3,7,25,33,34,38]
  No industry standards for data formats, range of values, and sample rates [34,38-40]
  Different devices use different algorithms for the same variable [3,7,38]
  Different type or placement of sensors on the body for the same variable [37]
  Different data definition for the same variable [7,33]

**Table 1:** Mappings between factors and intrinsic data quality challenges.

Factors influencing DQ^a^		Intrinsic DQ challenges		
		Completeness	Correctness	Heterogeneity
**Device- and technical-related factors**				
	Hardware issues [26,27]	✓^b^	✓	—^c^
	Software issues [24,25,27,29,34,35]	—	✓	✓
	Network and Bluetooth issues [29-31,34,36]	✓	—	—
**User-related factors**				
	User nonwear [7,24,26,30,33,34,36]	✓	—	—
	User error [27,29-31,33,34,37]	✓	✓	—
**Data governance-related factors**				
	Lack of standardization [3,7,25,33,34,38]	—	—	✓

^a^DQ: data quality.

^b^This indicates that the data quality challenge is associated with the factor according to the studies included in the review.

^c^Not available. This indicates that the data quality challenge was not particularly mentioned in studies as an associated challenge of the factor.

### Factors Affecting the Quality of Person-Generated Wearable Device Data

#### Device- and Technical-Related Factors

Device- and technical-related factors consist of issues related to (1) hardware, (2) software, and (3) network and Bluetooth. Issues related to hardware include sensor malfunction [26,29-32], the quality of sensors [3,7,24,32-34], and sensor degradation over time [27]. For instance, companies continuously upgrade their devices, which means that older models are outdated and may no longer be supported by the company [24]. This may affect studies that are interested in longitudinal data, as discontinued device support may lead to incomplete data [24].

There are several issues with software or algorithms used to interpret raw sensor data [24,25,27,29,34,35]. One major issue is that consumer wearables use proprietary algorithms for their devices [25,27,29,35]. Thus, it is difficult to know if or when consumer wearable companies change and update their algorithms [24]. The lack of transparency regarding the timing and impact of software change can impact data consistency between participants who have data from different periods and also between data from the same participant collected longitudinally [24].

Network and Bluetooth problems can also affect the data quality of wearable devices. Lack of wireless signals or lost satellite connections can cause errors and delays in capturing, synchronizing, and uploading the data [29,30,34,36]. In addition, the location tracking function might stop working when the user is in a building with poor satellite connection, which could lead to missing data problems [30].

#### User-Related Factors

A primary user-related factor is not wearing the device (nonwear time) [7,24,26,30,33,34,36]. Missing data that occur from nonwear is a major limitation to the accuracy of estimates derived from wearables because the pattern of missingness in these instances is often not at random (ie, missing not at random), which has implications for inferences that can be made based on these estimates [41,42]. Another user-related factor is incorrect use by users. For instance, researchers conducting time-sensitive studies should keep in mind that automatic time zone updates may fail, and users may forget to manually update or synchronize their time zone when traveling [29].

#### Data Governance-Related Factors

Data standard is an essential deliverable of data governance that can not only affect the comparability between data systems but can also influence the researcher’s ability to make reliable inferences from data [43]. However, wearable device data, more specifically consumer-grade wearables, are rarely standardized to interoperate with clinical systems, as such devices are developed for consumer use rather than research or clinical practice [44]. Lack of standardization can cause significant heterogeneity across data from different device brands (eg, Fitbit vs Garmin) or different models within the same brand (eg, Fitbit Charge 3 and Fitbit Inspire) and more broadly across individuals and different clinical centers. As a result, it might be difficult for researchers to integrate data sets and make a direct comparison between the analysis results from different device data [3,7,25,33].

### Intrinsic Data Quality Challenges of Person-Generated Wearable Device Data

One of the goals of this study was to identify potential data quality challenges when reusing data from the routine use of devices for research purposes. However, because of the lack of literature on the reuse of wearable device data, data quality challenges for research in general have been investigated. As a result of the review, three intrinsic data quality challenges were identified—completeness, correctness, and heterogeneity. Missing data were indicated as challenges occurring because of device malfunction, lost satellite connection or synchronization error, users not wearing the device, and devices unstably contacting the skin [7,26,30,34,36]. Incorrect data, which were more frequently stated as inaccurate data in studies, was another potential data quality challenge [26,27,33,35]. Poor sensor quality, the unknown limitations of proprietary algorithms, or user errors such as incorrect device placement can all contribute to incorrect data [26,33]. Another problem is the potential heterogeneity across data sources, which can lead to difficulty in intra- and intersubject comparisons [25,35,38]. This is because (1) companies do not always reveal whether or when they update their device algorithms or whether or when the users install the provided software updates, and (2) different devices may use different algorithms or data definitions for the same variable [25,35,38]. The focus of this study was on intrinsic data quality challenges, which are challenges on the data in its own right [21]. Thus, challenges extrinsic to data such as data accessibility, security, and privacy were not included.

## Discussion

### Principal Findings

Device- and technical-related, user-related, and data governance-related factors were identified as factors that influence the quality of wearable device data. These factors can potentially affect 3 intrinsic data quality challenges: completeness, correctness, and heterogeneity of data. Of note, the factors identified in this review are inherent to the characteristics of wearable device data as opposed to factors that could occur while processing the data, such as factors in extract, transform, and load (ETL) processes [45]. Researchers conducting multicenter studies should keep in mind that converting their wearable device data by using a common data model may induce additional errors during ETL processes [46].

Factors associated with data quality problems were classified into 3 main categories; however, the authors realized that the identified factors were highly connected to each other, and thus, the categorization could be subjective. For example, limited battery life is a device-related feature, but as a low battery level could make the user take off the device to charge the device, it was classified as a user-related factor. In addition, the proprietary algorithm of devices can be a data governance-related factor as proprietary algorithms lead to heterogeneity in multidevice data due to lack of data standards. However, the proprietary algorithm of devices was classified as a device-related factor because algorithms are part of the device and can produce data heterogeneity in single-device data as well. Despite the subjective nature of this work, three researchers iteratively refined the categories until a consensus was reached. As this is an early attempt to investigate data quality challenges for wearable device data, the authors expect this categorization to be refined in the future as researchers start to apply this framework while assessing data quality.

### Implications and Recommendations for Researchers

#### Summary of Recommendations for Researchers

Our study results indicate that a multitude of intrinsic data quality challenges exist for person-generated wearable device data, and we summarize the factors that underlie these challenges. We report completeness, correctness, and heterogeneity of data as the 3 primary concerns for researchers looking to conduct research using data from wearable devices. The implications and recommendations provided in this section are derived from the authors’ domain expertise and are based on existing literature both within and outside this review. A summary of the recommendations is presented in Textbox 2.

**Summary of intrinsic data quality challenges and recommendations for researchers.**

**Completeness**

- Report the definition of completeness used in research studies.
- Best practices on fitness-for-use measures for data completeness should be investigated.

**Correctness**

- Community effort to create a knowledge base of data quality rules is needed.
- Identify methods or external data sources that would help researchers retrospectively assess the plausibility of their data set.

**Heterogeneity**

- Data providers should collect metadata on which device brand, model, and software version the data are generated from.
- Researchers should check these metadata before conducting analyses and report it when publishing study results.

##### Data Completeness

Completeness is one of the major data quality challenges for wearable device data, mainly because users do not wear the device. Completeness is also a complex challenge, as various considerations need to be made by researchers to assess it. First, researchers need to determine how they would distinguish between true inactivity and device nonwear. This is especially the case for step count data, as missing data are unique in that they could appear as null values (eg, because of error in the device) or appear as zeros if the device is not worn. This is a challenge, as the cause of zero values (eg, nonwear, sedentary behavior, connectivity issue) is typically not documented, especially if the device is routinely used in daily lives. Previous studies have defined nonwear time with various thresholds for inactivity (zero count of activity) periods ranging from 10 to 60 minutes [47,48]. As different definitions of nonwear time may significantly change the total wear time per day and analysis results, reporting what threshold was used would be an important step for researchers [47].

In addition, there are multiple measures to consider when assessing data completeness among which one is valid day—a day with sufficient data that can be kept for analyses [49]. Tang et al [49] proposed three heuristic criteria for valid days: (1) minimum step count (eg, a day is valid if the daily step count is greater than 500), (2) the minimum count of hours with data (eg, a day is valid if there are 10 hours of data each with at least one step), and (3) 3-a-day (eg, a day is valid if there is data within 3 periods of the day).

In the past, research-grade devices did not have the capacity to collect data over time, but with the advent of newer devices that can collect data longitudinally over several months and years, concepts of valid week or valid month have been introduced. Researchers should question, for example, how many valid days per week or month is sufficient for their specific analysis; whether valid days, weeks, or months should be consecutive and for how long; or whether valid data should be regularly occurring rather than having long-term gaps in between valid data points. All these are fitness-for-use measures unique to person-generated wearable device data, which means that depending on the research question and data type involved, the definitions for valid days, weeks, and months may differ or may not be required. The large number of potential research questions and different data types makes a one-size-fits-all approach infeasible for data completeness and suggests the need to investigate fitness-for-use measures that apply to person-generated wearable device data. Furthermore, explicitly stating the completeness definitions used in the analyses would benefit future researchers in reproducing the work. As data completeness is complex in nature, further work to assist the assessment of data completeness would alleviate the burden on researchers.

##### Data Correctness

Checking the correctness of data values is another quality-related challenge, as it is impossible to retrospectively identify the correct value. This is especially the case for data generated through the routine use of wearable devices because it is unlikely that a gold standard data set would exist. One approach to circumvent this challenge might be to identify outliers that are against common sense and rules for plausibility based on published values in the literature. An example rule would be that there should be no steps counted during sleep mode. The fact that researchers are currently using ad hoc rules can lead to inconsistencies and difficulty in replicating studies. Thus, a community effort to create a knowledge base for data quality rules would be beneficial to researchers because creating data quality rules is time consuming and heavily dependent on domain experts. Another indirect method to speculate data correctness would be to assess the concordance of user input data, such as age, gender, height, and weight, with another data source such as the EHR. It is known that incorrect user input while setting up the device may result in incorrect data values, as there are variables calculated based on user input (eg, calorie expenditure) [50]. If the demographic data recorded in the wearable device and the EHR agree with each other, we can at least be assured that the data values were calculated based on a trustable user input. This is an important step for those who are interested in using both wearable device data and EHR data in their study.

##### Data Heterogeneity

**Figure 2:**
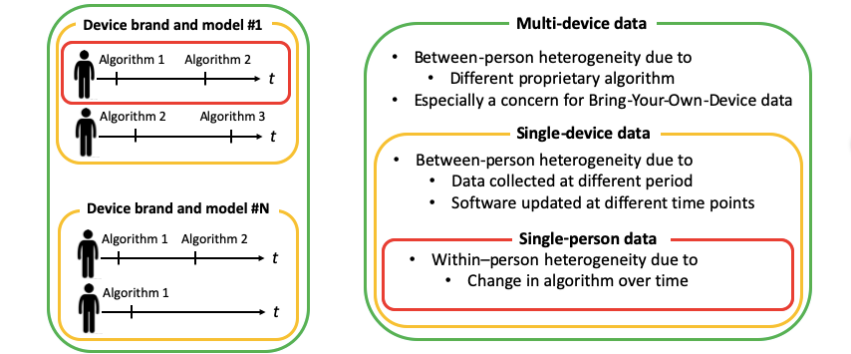
Data heterogeneity on three levels.

Through this review, the authors found that heterogeneity of data exists at three levels—single-person data (a data set generated by a single person), single-device data (data set generated by multiple people who use the same brand, model, and version of device; eg, a data set consisting of data generated from Fitbit Charge HR), and multidevice data (a data set generated by multiple people who use diverse brands, models, and versions of devices, eg, data set consisting of data generated from Fitbit Charge HR, Fitbit Alta HR, Withings Steel HR Sport, Apple Watch Series 3, etc). Figure 2 depicts the three levels of data heterogeneity.

In single-person data, a change in algorithms over time may produce within-person heterogeneity [24]. For single-device data, there would be between-person heterogeneity, as data are collected from multiple people at different periods, where different versions of algorithms can be used across people depending on the period of data collection [24]. Even if data are collected in the same period, heterogeneity could exist if the software is updated at different time points across individuals. In this setting, both between-person and within-person heterogeneity can occur simultaneously. For multidevice data, the heterogeneity increases even more because of the different proprietary algorithms used for different devices. There would be between-person heterogeneity across data from individuals using different devices in addition to the between-person heterogeneity across data from individuals using the same device and within-person heterogeneity across data from different time points within the same person. This would especially be a concern for data sets collected under a bring-your-own-device policy, as individuals would provide data from different device brands, models, and different periods. Thus, it is recommended that data providers collect metadata on which device brand, model, and software version the data are generated from, and researchers should check this metadata before conducting their analyses. It would also be a good practice to report these data when publishing study results so that they could be compared with other studies [51,52].

Through the literature review process, we found that there is a lack of studies that thoroughly investigate the data quality challenges of person-generated wearable device data, especially for research purposes. Although the current literature describes the existence of data quality problems, it rarely elaborates on how the data quality metrics were defined or how the data quality problems of wearable device data were assessed. For large-scale, routinely collected wearable device data that are commonly used for biomedical research, further studies are needed to deeply understand the data quality challenges for wearable device data and provide guidance to researchers.

### Limitations

One limitation of this study is that only one researcher went through the process of screening the title and abstract of studies. Therefore, the selection of literature could have been subjective in the initial phase of screening, and there is the possibility that some factors or challenges were not extracted because of potential biases in selecting the literature. However, the reviewer followed the systematic, a priori–defined selection criteria and data extraction rules to ensure consistency and reproducibility [53]. Although the initial screening of the literature was performed by a single author, other activities such as full-text screening, determining search queries, and categorizing extracted data were conducted by multiple authors. Another limitation is that although we excluded device validation studies in our review, these studies may mention factors affecting data quality for research. However, our full-text screening contained a few device validation studies, and we did not find unique information that was not captured from the final list of 19 studies.

### Conclusions

The goals of this review were to (1) summarize the factors associated with data quality reported in the literature with respect to passive data collection methods using wearable devices, (2) identify data quality challenges of wearable device data, and (3) deduce implications on data quality challenges for using data for research purposes. With this goal in mind, we identified three categories—namely device- and technical-related, user-related, and data governance-related factors—along with the associated data quality problems mentioned in the literature—namely completeness, correctness, and heterogeneity. In the case of the secondary use of data, knowing the factors may not directly help researchers, as most of the problems cannot be retrospectively amended. However, the value of this study is that it facilitates the understanding of the potential causes of data quality challenges, which is a complex and time-consuming process that requires thorough discussions among domain experts, analysts, and researchers [45,54]. Moreover, it could guide the application of appropriate analytical procedures to mitigate the negative impact on analytic results. Our review provides some insight into potential data quality problems, such as the incorrectness, incompleteness, and heterogeneity of data. However, further work is required to gain a deeper understanding of each challenge, to investigate if there are any other existing challenges that have not been discovered in the literature, and to provide guidance on data quality assessments for person-generated wearable device data.

## Appendix

Multimedia Appendix 1 Search terms used in scholarly databases and a summary of studies included in this review.

## Abbreviations

ACM: Association for Computing Machinery
EHR: electronic health record
ETL: extract, transform, and load
IEEE: Institute of Electrical and Electronics Engineers
NIH: National Institutes of Health
PGHD: person-generated health data
