# Supplementary material for: Factors Affecting the Quality of Person-Generated Wearable Device Data and Associated Challenges: Rapid Systematic Review
Source: JMIR Mhealth Uhealth. 2021 Mar 19;9(3):e20738. doi: 10.2196/20738 (PMC8294465; doi:10.2196/20738)
Supplement: Multimedia Appendix 1 [file mhealth_v9i3e20738_app1.docx]

**Table S1. Search terms used in scholarly databases**

| PubMed | ("Wearable Electronic Devices"[Mesh] OR "Fitness Trackers"[Mesh] OR wearable device[tiab] OR wearable technology[tiab] OR consumer wearable[tiab]) AND ("Data Accuracy"[Mesh] OR data quality[tiab] OR completeness[tiab] OR accuracy [tiab] OR correctness [tiab] OR consistency[tiab] OR currency[tiab] OR reliability[tiab] OR validity[tiab] OR integrity [tiab] OR data error[tiab]) |
| --- | --- |
| ACM | ("data quality" OR "data completeness" OR "data accuracy" OR "data consistency" OR "data integrity" OR "data correctness" OR "data currency" OR "data reliability" OR "data validity" OR "data error") AND ("wearable technology" OR "wearable device" OR "fitness tracker" OR "sensor data" OR "consumer wearable") |
| IEEE | ("data quality" OR "data completeness" OR "data accuracy" OR "data consistency" OR "data integrity" OR "data correctness" OR "data currency" OR "data reliability" OR "data validity" OR "data error") AND ("wearable technology" OR "wearable device" OR "fitness tracker" OR "consumer wearable") |
| Google Scholar | 1. (wearable device + "data quality")  2. (fitness tracker + "data quality")  3. (wearable technology + "data quality")  4. (consumer wearable + "data quality") |

**Table S2. Summary of studies included in the review**

| **Author** | **Topic** | **Type of Device or Data** | **Factors influencing Data Quality (DQ)** | **Potential intrinsic**  **DQ Challenges** |
| --- | --- | --- | --- | --- |
| Beukenhorst et al. (2018) [1] | Examines (a) the feasibility of using consumer  smartwatches (apps) to collect patient-reported outcomes alongside continuous sensor  data (physical activity data), and (b) the relationship between pain and physical activity. Focuses on  preliminary results of user engagement with the smartwatch app and data completeness. | Huawei Watch 2 with an app named ‘Koalap’, which collects patient-reported outcome and physical activity related sensor data | - Hampered uploading for example due to lack of cellular signal at home - Technical issue (transmission error) - Non-wear due to low battery life - Limited access to raw data | - Completeness |
| Bietz et al. (2015) [2] | Investigates the barriers of using person-generated health data for research. | Device unspecified (Self-tracked data generated through technologies such as mobile apps and wearable devices) | - Lack of device validation - Proprietary algorithm – difficult to interpret data and establish validity of data and replicate research - Lack of standardization | - Correctness - Heterogeneity |
| Cleland et al. (2018) [3] | Describes the process of collecting large scale, diverse activity recognition data. Analyzes the feasibility of using the data for building activity recognition models. | Shimmer wireless sensor platform | - Poor calibration by users - Variations in data collection methodology by individuals | - Correctness (Outliers) - Heterogeneity |
| Codella et al. (2018) [4] | Describes authors' experience/challenges in working with PGHD as a data analyst. Reviews literature to identify stakeholders' concerns on PGHD and mapping it to an existing data quality framework. | General PGHD including mobile apps and wearable technology | - Lack of validation of consumer devices - Users do not feel incentivized to continuously/consistently use devices - Poor battery performance - Devices are bulky, uncomfortable - Using different device/models   - Various formulas exist for calculating energy expenditure   - Assumptions not known on how sleep measures are inferred - Lack of standardization for data collection and formatting - Difficulty in accessing raw data needed for research | - Completeness - Heterogeneity - Correctness |
| Constantinou et al. (2017) [5] | Explores the feasibility of using consumer activity monitor data for marathon research. | Fitbit consumer activity monitors | - Hardware failures - Software issues - Variation in data validity and reliability between different models - Lack of internet connectivity or synchronization errors - Incorrect device input by users (Forget to update/sync time zone when travelling) - Data access issue for more granular data (important problem but is an extrinsic data quality issue, so it was excluded in our results) | - Completeness - Correctness - Heterogeneity |
| Fawcett et al. (2015) [6] | Explores challenges of discovering knowledge from quantified-self data through data mining. | A 6-month worth dataset of a single person consisting of 15 variables collected from mobile apps, wearables (Bodymedia), and self-reported data | - Forget to wear devices (non-wear) - Faults in sensors (sensor errors) | - Completeness - Correctness |
| Duking et al. (2018) [7] | Provides recommendations on the evaluation of trustworthiness (reliability & validity) of data provided by wearables. | Wearable sensors for physical activity monitoring | - Old device - Blackbox algorithm – no access to raw data - Inaccurate positioning on body | - Correctness (Unreliable data) |
| Hardy et al. (2018) [8] | Compares user acceptance on location-tracking technologies among chronically ill population and discusses its implications for research. | Location-enabled smartphone, GPS watch/activity tracker | - Device malfunction - Timely response to lost satellite connection - Synchronization delay or error - User non-wear/selective use   - Short term charging issues   - Patient health issues   - Unsatisfied with the appearance of device   - Selectively decide not to use on specific whole days or for certain activities   - Concerns on data privacy/security   - Poor usability experience | - Completeness - Correctness |
| Julicher et al. (2018) [9] | Provides an overview on wearables and its legal and social implications. | Wearables in general | - Proprietary systems to collect and process data – leads to interoperability issues | - Correctness - Heterogeneity |
| Liang et al. (2017) [10] | Investigates sources of measurement error of Fitbit self-tracking data. | Fitbit sleep tracking data | - Sensor and algorithm limitations (does not have the capability to correctly measure metrics) - Not wear device regularly - Lack of data definition (sleep metrics) | - Correctness - Heterogeneity |
| Mezghani et al. (2015) [11] | Describes a generic  semantic big data architecture addressing (1) data heterogeneity  by proposing the Wearable Healthcare Ontology, and (2)  scalability by adopting the NIST Big Data reference architecture  and storing the wearable data into distributed clusters  deployed in a cloud environment. | Wearables for healthcare | - Heterogeneous data format and algorithm encoding transmitted data for wearable data coming from multiple sources | - Heterogeneity |
| Reinerman-Jones et al. (2017) [12] | Discusses considerations when using a fitness tracker for psychophysiology  research. Compares data collected from the Microsoft Band 2 to two different  FDA approved medical grade ECG devices to validate the utility of using Microsoft Band 2 for scientific research. | Fitness tracker in general and a specific example with Microsoft Band 2 | - Sensor types, capabilities, and sampling rates - Software updates may change settings to default setting - Bluetooth or Wi-Fi connectivity - Data upload delay - Battery life - Tracker incorrect tautness & shifts around (especially, optical HR sensor) - Device placement and sensor orientation can be inconsistent across users - No industry standards for data formats, sample rates, and sensor reporting units | - Completeness - Correctness - Heterogeneity |
| Wood et al. (2015) [13] | Reviews considerations, barriers, and limitations for integrating PGHD to clinical trial and research. | PGHD in general (including passively collected sensor data, user-entered data) | - Device accuracy/validity unclear - Different device/models may measure metrics differently so the results may not be interchangeable (no standards) | - Correctness - Heterogeneity |
| Wright et al. (2017) [14] | Reviews opportunities, limitations, and challenges for using consumer fitness trackers in research. Examines use cases of fitness trackers in research. | Consumer physical activity monitors | - Lack of device validity - Unknown algorithm - Basic adherence - Comfort - Battery life - Misplacement of device | - Completeness - Correctness |
| Banerjee et al. (2017) [15] | Discusses data quality challenges and solutions for IoT systems based on two use cases. | IoT devices in general (Hexoskin, Smart home system) | - Error from user entry - Data corruption, failed sensor - Sensor (algorithm) accuracy in a certain population - Network connectivity issues | - Completeness - Correctness |
| Guo et al. (2017) [16] | Proposes context-aware scheduler, a system that dynamically adjusts data collection schedule to gather personal data from multiple wearables. | Context aware scheduling system for Wear-I, a wearable system that uses multiple devices to collect personal from embedded sensors in the wearables | - Hardware limitations (battery life, CPUs, networks) | - Completeness - Heterogeneity |
| Karkouch et al. (2016) [17] | Overviews data quality and data quality dimensions for IoT data. Discusses factors affecting IoT data quality and their impact on data quality problems, techniques to improve data quality. | IoT devices in general | - Lack of power and storage - Intermittent loss of network - Lack of precision in sensor - Sensor error | - Completeness (Dropped readings) - Correctness (Duplicate data; Unreliable readings) - Heterogeneity (Multi-source data inconsistencies; Multi-source data time alignment) |
| Lai et al. (2017) [18] | Reviews literature on consumer health informatics and patient-generated health data to identify topics dealt and the existing gap in PGHD-related articles. | PGHD in general (including wearable devices) | - Lack of standard data models | - Heterogeneity |
| Oh et al. (2015) [19] | Explores user experience issues of quantified-self tools by analyzing user reviews. | Various quantified-self tools (Withings, Fitbit, etc.) | - Heterogeneous units and value ranges when integrating different data sources | - Heterogeneity |

**References**

1. Beukenhorst A, Sergeant J, Little M, McBeth J, Dixon W. Consumer Smartwatches for Collecting Self-Report and Sensor Data: App Design and Engagement. Stud Heal Technol Inf. 2018;247:291–295. PMID: 29677969

2. Bietz M, Bloss C, Calvert S, Godino J, Gregory J, Claffey M, et al. Opportunities and challenges in the use of personal health data for health research. J Am Med Inf Assoc. 2016;23:e42–e48. PMID: 26335984

3. Cleland I, Donnelly M, Nugent C, Hallberg J, Espinilla M, Garcia-Constantino M. Collection of a Diverse, Realistic and Annotated Dataset for Wearable Activity Recognition. 2018 IEEE Int Conf Pervasive Comput Commun Work. 2018;

4. Codella J, Partovian C, Chang H-Y, Chen C-H. Data quality challenges for person-generated health and wellness data. IBM J Res Dev. IBM; 2018;62(1):1–3.

5. Constantinou V, Felber AE, Chan JL. Applicability of consumer activity monitor data in marathon events: an exploratory study. J Med Eng Technol. England; 2017;41(7):534–540. PMID: 28954563

6. Fawcett T. Mining the quantified self: personal knowledge discovery as a challenge for data science. Big Data. 2015;3(4):249–66.

7. Düking P, Fuss F, Holmberg H, Sperlich B. Recommendations for assessment of the reliability, sensitivity, and validity of data provided by wearable sensors designed for monitoring physical activity. JMIR MHEALTH UHEALTH. 2018;6(4). PMID: 29712629

8. Hardy J, Veinot TC, Yan X, Berrocal V, Clarke P, Goodspeed R, et al. User acceptance of location-tracking technologies in health research: implications for study design and data quality. J Biomed Inform. 2018;79:7–19. PMID: 29355784

9. Jülicher T, Delisle M. Step into “The Circle”—A Close Look at Wearables and Quantified Self. Big Data Context. 2018. p. 81–91.

10. Liang Z, Ploderer B, Chapa-Martell MA. Is Fitbit Fit for Sleep-tracking?: Sources of Measurement Errors and Proposed Countermeasures. Proc 11th EAI Int Conf Pervasive Comput Technol Healthc. New York, NY, USA: ACM; 2017. p. 476–479.

11. Mezghani E, Exposito E, Drira K, Da Silveira M, Pruski C. A semantic big data platform for integrating heterogeneous wearable data in healthcare. J Med Syst. 2015;39(12). PMID: 26490143

12. Reinerman-Jones L, Harris J, Watson A. Considerations for using fitness trackers in psychophysiology research. Hum Interface Manag Inf Information, Knowl Interact Des. 2017. p. 598–606.

13. Wood WA, Bennett A V, Basch E. Emerging uses of patient generated health data in clinical research. Mol Oncol. Wiley Online Library; 2015;9(5):1018–1024. PMID: 25248998

14. Wright SP, Hall Brown TS, Collier SR, Sandberg K. How consumer physical activity monitors could transform human physiology research. Am J Physiol Regul Integr Comp Physiol. United States; 2017;312(3):R358–R367. PMID: 28052867

15. Banerjee T, Systems AS. Iot quality control for data and application needs. IEEE Intell Syst. 2017;

16. Guo A, Access JM. Context-aware scheduling in personal data collection from multiple wearable devices. IEEE Access. 2017;5.

17. Karkouch A, Mousannif H, Al Moatassime H, Noel T. Data quality in internet of things: A state-of-the-art survey. J Netw Comput Appl. Elsevier; 2016;73:57–81.

18. Lai A, Hsueh P, Choi Y, Austin R. Present and future trends in consumer health informatics and patient-generated health data. Yearb Med Inf. 2017;26(1):152–159. PMID: 29063559

19. Oh J, Lee U. Exploring UX issues in Quantified Self technologies. 2015 Eighth Int Conf Mob Comput Ubiquitous Netw. 2015. p. 53–59.
